# The influence of glucose intake and psychosocial stress on food craving in healthy adults

**DOI:** 10.1016/j.cpnec.2026.100372

**Published:** 2026-08-05

**Authors:** Flora Nasilowski, Julia Reichenberger, Bernadette Denk, Raphaela Gaertner, Elea Klink, Eva Unternaehrer, Nina Volkmer, Stella Wienhold, Jens Pruessner, Maria Meier

**Affiliations:** a Department of Psychology, University of Konstanz, Konstanz, Germany; b Department of Psychology, Centre for Cognitive Neuroscience, Paris-Lodron-University of Salzburg, Salzburg, Austria; c Centre for the Advanced Study of Collective Behavior, Konstanz, Germany; d University Psychiatric Clinics Basel (UPK), University of Basel, Basel, Switzerland; e Department of Child and Adolescent Psychiatry/Psychotherapy, University of Ulm, Germany

**Keywords:** Food craving, Stress, Cortisol, Trier social stress test, Blood glucose

## Abstract

Stress has been linked to the consumption of palatable food and may contribute to the development of obesity. Interindividual differences in cortisol stress reactivity are discussed as mediating mechanisms, yet findings linking food craving to cortisol reactivity are heterogeneous. Methodological differences in whether participants receive a sugary preload or are invited fasted may contribute to these divergences. Hence, this study set out to systematically investigate the effect of glucose and psychosocial stress on food craving in healthy adults.

Overall, 62 healthy, fasted adults (mean age = 23.13; *SD* = 3.02; 54.84 % female, 45.16 % male) reported current *food craving* on the Food Craving Questionnaire, received either *glucose* or *water* and were later exposed to psychosocial *stress* or a *friendly control condition*. After stressor exposure, food craving was assessed a second time. We repeatedly assessed salivary cortisol and blood glucose and used linear mixed models to test whether the experimental manipulations predicted changes in craving. We further explored associations with sex and stress-eating tendency.

Blood glucose increased in those consuming glucose and the stress test increased cortisol significantly. Higher cortisol reactivity, and a greater increase in blood glucose led to significant reductions in food craving over time. We found no supportive evidence that glucose and cortisol reactivity interactively affected craving.

These findings suggest that strong metabolic signals triggered by calorie intake may be more influential than cortisol in shaping immediate food craving in moderate, acute stress situations. Additionally, acute cortisol elevations may not universally promote food craving.

## Highlights

• Higher cortisol reactivity was associated with reductions in food craving over time.
• Higher glucose increases were linked to reductions in food craving over time.
• We found no evidence for a significant interaction between glucose and cortisol reactivity to predict craving.
• The glucose manipulation was more strongly associated with food craving following acute stress than cortisol responses.

## 1 Introduction

Stress is linked to the consumption of highly palatable food and clinical and subclinical forms of overeating [[1], [2], [3]]. From an evolutionary perspective, stress-response systems evolved to mobilize energy in response to acute physical threats. In modern environments, however, stressors are predominantly chronic and psychological, such that repeated energy mobilization without corresponding expenditure may promote positive energy balance and fat accumulation over time [4]. Indeed, longitudinal and epidemiological studies link chronic stress to weight gain and a higher body mass index [[5], [6], [7], [8]]. As such, stress likely contributes to the development of obesity and other metabolic disorders [9]. Yet, individuals differ markedly in their eating responses to stress, with approximately 40 % reporting increased food intake, a comparable proportion reporting no change, and a minority reporting reduced intake [[5], [6], [7], [8]]. Understanding the physiological and psychological mechanisms that underlie these interindividual differences is critical to better understand risk factors for stress-related overeating as targets for prevention programs.

One mechanism that may contribute to the variability in stress-related eating is the activation of the hypothalamic-pituitary-adrenal (HPA) axis. When ingesting food, the rising blood glucose level triggers insulin release, which promotes satiety [10]. However, stress may disrupt this balance. Acute stress triggers cortisol release via hypothalamic and pituitary signaling, preparing the body to meet increased energy demands by promoting gluconeogenesis and the breakdown of energy stores, ensuring sufficient glucose supply for cardiovascular, muscular, and cognitive activity [11,12]. Yet, cortisol impairs insulin signaling and stimulates glucagon release, overriding appetite-suppressing effects and increasing the drive to consume palatable foods [11,13,14]. As such, stress-related hormonal changes may override insulin's appetite-suppressing effects. In addition, cortisol also stimulates the mesolimbic dopamine pathway, which is implicated in reward-seeking behavior and may thereby increase the hedonic value of food [11,12]. Indeed, administration of medical doses of glucocorticoids increases *ad libitum* energy intake in healthy men over the course of a week [15], directly demonstrating cortisol's impact on appetite. Taken together, heightened cortisol reactivity may be a central pathway linking stress exposure to increased food intake independent of physiological hunger [16]. As changes in cortisol levels from baseline to post-stress exposure, i.e., cortisol stress reactivity, varies considerably between individuals and across different stressors [17], this interindividual variability may contribute to individual differences in the vulnerability to stress-related eating and obesity [2]. Importantly, chronic stress may not only influence immediate food intake but can also have longer-term metabolic implications, as higher endogenous cortisol production and free cortisol levels have been linked to increased visceral fat and lower insulin sensitivity [18]. Additional pathways that interact with cortisol in this context comprise elevated levels of ghrelin and impaired leptin signaling [19,20], which may jointly contribute to a disruption in energy balance [21] by reducing energy expenditure and altering the gut microbiota and gut–brain signaling which overall promote weight gain [11].

To date, several studies examined the link between cortisol stress reactivity and stress-induced eating, but findings are mixed. For example, women with higher cortisol reactivity have been observed to consume more calories following stress compared to low reactors [2]. In line with this, women with higher cortisol stress reactivity and more daily hassles showed increased snack intake over two-weeks [22]. Yet higher cortisol levels after stress have also been associated with decreased snack intake in obese women, with no such relation being observed in lean women [23]. At the same time, lower cortisol reactivity was associated with greater hunger in a healthy mixed-sex sample [24] and with higher post-stress food intake among chronically stressed women [25]. Together, these heterogeneous findings suggest substantial interindividual variability in the vulnerability to stress-related eating [6].

While parts of this heterogeneity may be related to risk factors such as chronic stress and weight status [11], there are also methodological differences that may explain these inconsistencies, particularly regarding participants' energy preload and nutritional state before stress exposure. While some studies provided standardized meals [25], others offered a snack or grape juice drink an hour before testing [2,24], and some instructed participants to fast for several hours [26] or overnight [23]. Such variation in energy preload likely led to differences in hunger, satiety and blood glucose at the onset of the stressor, potentially influencing both food craving and the cortisol stress response. While hunger and satiety at baseline have often been considered, the potential influence of blood glucose and effects of glucose preloads (i.e., offering snacks or sugary drinks) on stress-induced food craving have hardly been accounted for, although there is considerable evidence supporting that both factors may play a role in this context. First, glucose intake prior to stress robustly enhances the cortisol stress response [27], which may in turn modulate post-stress food craving. Second, glucose preloads may directly affect food craving. While previous research found no change in food craving after glucose vs. water infusion to the stomach [28] this invasive treatment circumvents that participants taste what they consume. In the aforementioned experiments [2,24,25], participants consumed a meal, snack or juice orally. Under these circumstances, individuals typically try to infer a meal's or drink's content based on apparent features such as color, verbal descriptions or taste. As such, sugary preloads may influence expectations on whether a meal or drink provides energy or not, which may in turn affect food-related evaluations and cravings. Previous research indicates that explicit, verbal descriptions of drinks can infer with their effect on subsequent food craving, e.g. when a milkshake is labelled as healthy (snacking is increased) vs. not (snacking is reduced) [29]. It is likely that similar processes may be triggered implicitly, e.g. based on a drink's sweet taste. Nevertheless, the effect of glucose preloads and glucose reactivity, i.e., the change in blood glucose levels from baseline to peak levels following a sugary drink, on stress-induced food craving has not been examined systematically to date.

The present secondary analysis therefore aimed to examine the effect of glucose preload on the association between cortisol reactivity and changes in food craving following stress exposure. Participants arrived in the laboratory session after a 5-h fast and consumed either a *glucose* preload or *water* before undergoing a modified version of the Trier Social Stress Test (TSST) [30] or a friendly control TSST [31]. Additionally, they reported food craving before the drink, and after the TSST. We expected that participants exposed to stress would exhibit a greater increase in self-reported food craving as compared to those in the friendly control condition (H1a) and that a higher cortisol reactivity would predict higher increases in food craving (H1b). Second, we expected that glucose intake would modulate stress-related food craving. Specifically, we hypothesized that glucose intake reduces food craving in the friendly control condition, while it increases food craving in the stress condition (stress × drink interaction effect, H2a). Analogously, we expected that glucose reactivity is negatively associated with food craving at low cortisol reactivity, while it is positively associated with food craving at high cortisol reactivity (cortisol reactivity x glucose reactivity interaction effect, H2b). All analyses were adjusted for effects of stress eating tendencies. In an exploratory manner, we preregistered to evaluate potential sex-specific effects in the confirmatory analyses and tested associations between different aspects of food craving (e.g., positive reinforcement of food, physiological hunger, desire to eat) and cortisol and glucose reactivity.

## 2 Methods

This is a secondary analysis of an existing dataset from a project that focused on the effect of glucose and stress on long-term memory [32]. Whereas the original study focused on object and word retrieval as primary outcomes, the present study addresses a conceptually distinct question by examining appetitive processes, specifically short-term changes in food craving following glucose consumption and psychosocial stress. While there is some overlap concerning manipulation checks, we defined distinct hypotheses targeting outcomes related to food craving in the current analysis. Those are grounded in a distinct theoretical framework related to stress-related eating and metabolic regulation. Data collection took place at the University of Konstanz, Germany, from December 2022 to August 2023. The experimental procedure adhered to the Declaration of Helsinki and was approved by the Ethics committee of the University of Konstanz (IRB 45/2022). Participants signed a written informed consent prior to participation and were reimbursed with either study credits or monetary compensation (40 €).

### 2.1 Participants

Healthy participants aged 18 or older, with sufficient proficiency in German, were recruited through flyers distributed on campus and in the city of Konstanz, as well as via the University's online participant database (SONA Systems). Study eligibility was assessed through a 10-min online survey. We controlled for factors known to influence the autonomic and endocrine stress response [33]. To do so, the following exclusion criteria were applied: (1) body mass index below 18.5 or above 30 kg/m^2^, (2) heavy smokers (>5 cigarettes per day), (3) disturbed circadian rhythm due to night shift or jetlag, (4) mental or somatic disorder(s), (5) medication intake, (6) alcohol or drug abuse, (7) clinically relevant symptoms of depression (based on Beck's Depression Inventory II sum score >18 [34]). Further, female participants were excluded if they reported an irregular, very short (<21 days) or long (>37 days) menstrual cycle, current pregnancy, being in menopause, or using hormonal contraceptives [35]. Psychology students in the fifth semester or higher were excluded due to high chances of prior knowledge of the stressor.

Overall, 62 healthy, fasted adults (mean age = 23.13; *SD* = 3.02; 54.84 % female, 45.16 % male) participated in the study. Participants were instructed to arrive at the laboratory in the afternoon (start at 1.30 p.m. to control for the HPA axis’ diurnal rhythm) in a fasted state, having abstained from food and caloric beverages for at least 5 h. They were asked to refrain from consuming alcohol for 24 h before testing, and to avoid caffeinated beverages, chewing gum, or consuming nicotine for at least 2 h before testing [33,36]. Additionally, participants were advised to maintain a regular sleep schedule and to avoid strenuous physical activity on the evening before and the morning of the session [36].

### 2.2 Experimental procedure

The study had a mixed design with the between-subject factors *drink* (water, glucose) and *stress* (friendly control, stress), and the repeated-measure within-subject factor *time* (psychophysiological stress measures, food craving). Independent of sex, participants were randomly assigned to the four groups (*drink* x *stress*) based on a list created with the R package *randomizr* [37] and the function *complete_ra* (.). The resulting group sizes were the following: (1) water, friendly control (*n* = 14), (2) glucose, friendly control (*n* = 16), (3) water, stress (*n* = 18), (4) glucose, stress (*n* = 14).

**Fig. 1:**
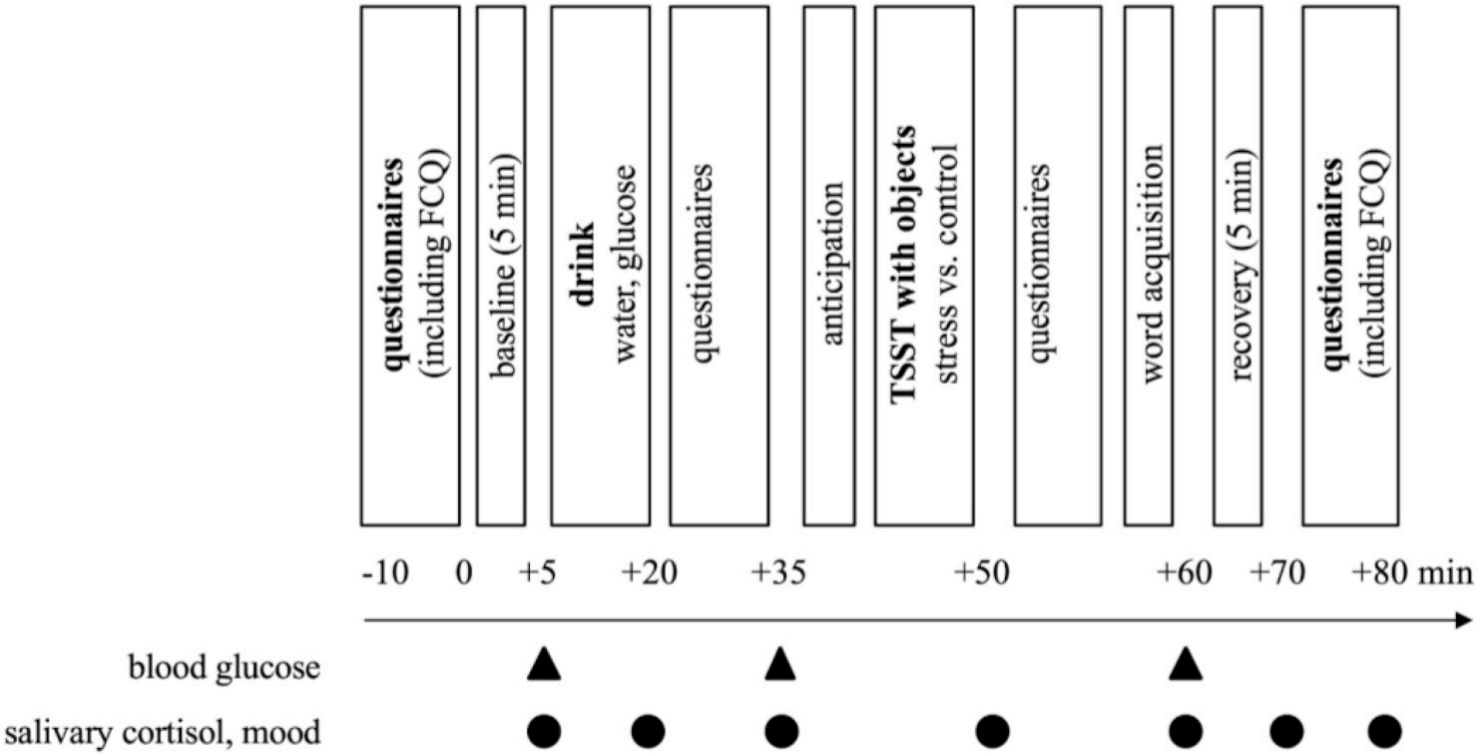
Experimental procedure. FCQ = Food Craving Questionnaire (state version). TSST = Trier Social Stress Test.

The experimental sessions of the main experiment were performed on two consecutive days in the afternoon between 1.30 and 5 p.m. to control for circadian effects observed in HPA axis activity [38] and metabolism [39]. On day 1, participants completed the stress test and on day 2, they completed memory tests. The session lasted approximately 90 min on day 1 and 45 min on day 2. As the data from the second laboratory visit are not relevant to the current analysis, the interested reader is referred to previous work in which the full procedure is explained in greater detail [32]. The procedure of the study parts relevant to this analysis is depicted in Fig. 1.

Eligible participants were invited to the laboratory after a 5-h fast, as postprandial glucose concentrations may take up to 5 h to return to baseline [40]. Upon arrival at the laboratory, they were equipped with an electrocardiogram and filled in a short questionnaire, which included the Food Craving Questionnaire (state version [41]). After that, a physiological baseline was recorded (sitting in silence with eyes open for electrocardiography recording, not relevant to this project). Then, they consumed the assigned drink (*glucose* or *water*). During an absorbance period of approximately 25 min that ensured sufficient time for glucose absorption into the bloodstream [42], participants completed questionnaires (including the Salzburg Stress Eating Scale [43]). Then, they either underwent the TSST (stress condition) or the friendly TSST (friendly control condition; [30,31]). Later, at the expected cortisol peak, participants were asked to memorize a list of words. After a recovery period that captured a physiological baseline post-stress, participants filled in further questionnaires, which again included the Food Craving Questionnaire (state version [41]).

We repeatedly collected saliva samples for the determination of salivary cortisol and subjective mood ratings on visual analog scales on seven prescheduled timepoints and assessed blood glucose concentrations on three timepoints throughout the study (see Fig. 1).

### 2.3 Experimental manipulations

#### 2.3.1 Drinks

Participants were asked to drink a glass of non-sparkling, cooled (4°C), mineral water (200 mL). In the *glucose* condition, 75 g of glucose were dissolved in the water. The amount of glucose was comparable to drinks used in previous work [[44], [45], [46], [47]]. Both participants and the experimenter were blinded to the drink content.

#### 2.3.2 Stress and friendly control condition

Acute stress was induced using the Trier Social Stress Test (TSST [48]), a widely established and standardized protocol for the experimental induction of psychosocial stress. The TSST reliably elicits a robust increase in both endocrine and autonomic stress markers [49]. The standard procedure consists of a 5-min anticipation phase during which participants are instructed to prepare for a mock job interview. This is followed by a 5-min public speaking task delivered in front of a video camera and two evaluators dressed in laboratory coats, who maintain a neutral and non-reinforcing demeanor. Immediately thereafter, participants complete a 5-min mental arithmetic task involving serial subtraction, during which they are required to restart after each error. As the primary aim of the experiment included testing effects of stress on memory [32], we used an adapted version of the TSST that includes 24 objects (e.g., mug, ruler) that are placed in the TSST room [30,50]. The judges interact with those objects in a standardized manner, and participants’ recognition memory of the objects is tested unexpectedly. In the TSST version used here, the public speaking task is prolonged to 10 min, and no arithmetic task is performed. The detailed protocol can be retrieved from the supplemental material of a previously published report [32].

In the control condition, the friendly version of the TSST with Objects was performed, which follows the same general procedure but lacks the video recording. During the speech part, participants are allowed to speak freely about personal topics such as career aspirations or hobbies, while the judges respond in a supportive and encouraging manner. The friendly version of the TSST typically does not activate the HPA axis in healthy men and women [31].

### 2.4 Measures

#### 2.4.1 Blood glucose

Blood glucose concentrations (in dg/mL) were measured three times from capillary blood of the fingertip using disposable lancets (Roche Diabetes Care, Mannheim, Germany) and a glucometer (GlucoMen Areo, A. Menarini diagnostics, Berlin, Germany): (1) as a baseline before drink consumption (+5min), (2) approximately half an hour after drink consumption, when blood glucose levels are expected to have risen considerably, and before the TSST (+35min), (3) approximately half an hour after the second reading and after the TSST at the estimated cortisol peak (+60min). In eight occasions, capillary blood sampling was unsuccessful due to insufficient blood volume, and glucose values could therefore not be determined. *Glucose reactivity* was calculated as the area under the glucose response curve with respect to increase (AUCi_gluc [51]) and used as a continuous predictor of changes in food craving.

#### 2.4.2 Salivary cortisol

Salivary samples were collected at seven timepoints in 10-15 min intervals using Cortisol Salivettes® (Nümbrecht, Germany): (1) at baseline (+5min), (2) after drink consumption (+20min), (3) directly before the TSST (+35min), (4) directly after the TSST (+50min), (5) at the estimated cortisol peak (+60min), and in 10 min intervals thereafter to capture the recovery (sample 6 at +70min and sample 7 at +80min). The samples were stored at −20°C until biochemical analysis in the laboratory of the working group Neuropsychology at the University of Konstanz. After thawing, samples were centrifuged at 2500 rpm for 10 min. Cortisol concentrations (in nmol/L) were determined in duplicate using a validated enzyme-linked immunosorbent assay (ELISA, RE52611, IBL international GmbH, Hamburg, Germany), designed for the quantitative analysis of free cortisol in human saliva. Intra- and inter-assay coefficients of variation (CV) were 13.40 % and 22.18 %. Four samples did not contain sufficient saliva for cortisol determination, and one sample yielded enough volume for only a single determination. Samples exhibiting a high inter-assay CV (>20 %) in the initial duplicate analysis were reassessed. To minimize potential bias arising from differences in assay batch, storage conditions, and repeated freeze-thaw cycles, the more plausible value from the original determination was retained as a single determination in the analysis. *Cortisol reactivity* was calculated as the area under the cortisol response curve with respect to increase (AUCi_cort [51]) and used as a continuous predictor of changes in food craving.

#### 2.4.3 Subjective stress

Concurrently with the collection of saliva samples, participants provided *subjective stress* ratings on visual analog scales (VAS) that ranged from 0 (not at all) to 100 (very). VAS data from three participants was missing due to technical issues. *Subjective stress reactivity* was calculated as the area under the subjective stress response curve with respect to increase (AUCi_stress [51]) and used as a continuous predictor of changes in food craving.

#### 2.4.4 Food craving

Participants filled in the Food Craving Questionnaire-State (FCQ) twice to measure the intensity of momentary *food craving* [52]: (1) immediately after arrival at the laboratory, and (2) in the questionnaire period following the estimated cortisol peak. We used the German version of the FCQ [41] which consists of 15 items and five subscales: “An intense *desire to eat*”, “Anticipation of *positive reinforcement* that may result from eating”, “Anticipation of *relief from negative states* and feelings as a result of eating” and “*Lack of control* over eating”, “*Craving* as a physiological state (i.e., hunger)”. A total score was computed by summing up all items (theoretical range: 15-75). Participants indicated the extent to which a statement applied to them on a Likert scale from 1 (*Strongly disagree*) to 5 (*Strongly agree*). In previous work, the FCQ showed excellent internal reliability (*α* > .90) and a low retest reliability of *r* < .60 [41,52,53]. In our sample internal consistency was excellent at both time points and Cronbach's alpha was α = .92 before, and α = .94 after the TSST. Data from one participant at the second timepoint was missing due to technical issues. In the statistical analysis, we focused on the change in the FCQ sum score, as recommended earlier [53]. For correlational analyses with *cortisol* and *glucose reactivity*, change scores for the total score and each subscale were computed by subtracting the pre-TSST level from the post-TSST level. For this *change in food craving*, a positive value represents an increase in food craving, while negative values present a decrease in food craving over time.

#### 2.4.5 Potential covariates

As preregistered, we considered self-reported demographic information (e.g., sex, age, body mass index [BMI]), the time since last meal, food craving before the TSST, baseline blood glucose levels, and cortisol at baseline as potential covariates in the statistical analysis as these variables have been linked to stress reactivity and food craving in the past [33,53]. Sex was assessed as self-reported sex assigned at birth (male, female, intersex). As some individuals tend to overeat and others tend to reduce their food intake in response to stress, we further included the self-reported tendency to change eating patterns in times of stress (*stress eating tendency*) using the Salzburg Stress Eating Scale (SSES [43]) in the list of potential covariates. The SSES comprises 10 items which are scored from 1 (*I eat much less than usual*) to 5 (*I eat much more than usual*). As suggested, we computed a mean score that indicated the tendency to eat less when stressed (score < 3), eat the same amount as usual (score = 3), or eat more when stressed (score > 3). In the present sample, internal consistency was good (α = .89), indicating strong reliability consistent with the initial validation study [43].

As previous studies have repeatedly reported chronic stress to be a modulator of the link between stress and eating, we additionally included perceived stress within the last month to the potential covariates (not preregistered). It was assessed using the Perceived Stress Scale [54] which comprises 10 items that are scored on a 5-point Likert scale that ranges from *never* (1) to *very often* (5). We computed a sum score to index *chronic stress.* In the present sample, internal consistency was good (α = .84).

### 2.5 Statistical analysis

The statistical analysis plan was registered on the Open Science Framework (OSF) on October 7, 2025 (https://doi.org/10.17605/OSF.IO/GMZ92). The analysis was performed with R version 4.4.0 (2024-04-24) in RStudio version 2025.5.0.496.

We checked for outliers, i.e., values that lie below or above 3*SD* from the mean of the sample, in the distributions of the variables used in the statistical models and applied winsorizing to decrease their impact on the statistical analyses (applied to 11 of the cortisol values [2 %] and one of the blood glucose values [<1 %]). FCQ data was available for all participants except one participant, who missed the post-stress FCQ score. This value was estimated based on the mean of the respective experimental condition. We estimated missing values in the physiological data (glucose, cortisol) based on the sample mean of the respective experimental condition.

For descriptive purposes, we tested whether the potential covariates (cf. section 2.4.5) differed significantly between the four study groups using ANOVAs. The list of variables was based on theoretical considerations [33,53] and included sex, age, BMI, fasting duration, food craving before the TSST, baseline blood glucose levels, and cortisol at baseline, stress-eating tendency (all preregistered) and chronic stress (not preregistered). As preregistered, we determined whether to include a potential covariate into the statistical analysis based on whether they were significantly associated with the outcome of our confirmatory analyses (i.e., changes in food craving). This combination of a theory-driven approach to covariate identification with a data-driven approach was used to address problems arising from overfitting and aligns with recommendations to only included covariates in the statistical analyses if they are associated with the outcome [55].

To test our confirmatory hypotheses, we used linear mixed models modeled with the R package *nlme* [56]. Specifically, we tested if changes in food craving over *time* (2 levels: pre- and post TSST) were predicted by *stress* condition (2 levels: friendly control TSST and stressful TSST; H1a) or *cortisol reactivity* (H1b). To test the second hypothesis, we added the interaction term *stress* and *drink* condition (2 levels: water and glucose; H2a) or *cortisol* and *glucose reactivity* (H2b) while controlling for *stress eating tendency*. To explore potential effects of *sex*, it was added to the models by including the main effect of *sex* as well as the interaction term *sex* x *time* x *stress* x *drink*. All models were built in a stepwise approach: We first added a random intercept for participants, random *time* slopes and a linear fixed effect of *time* to the linear mixed models. Then, we added the conditions of interest (*stress, drink* in H1a and H2a) or the continuous reactivity predictors, respectively (*cortisol reactivity, glucose reactivity in* H1b and H2b), before covarying for the interaction term *stress eating tendency x time*. In an exploratory manner, we tested the effect of the *sex × time* interaction effect. We evaluated the significance of the coefficients and computed marginal and conditional *R*^2^ with the package *performance* [57]. The respective model formulas for the linear mixed models can be obtained from the supplementary material (cf. Supplementary Table S1). Model assumptions (i.e., linearity, absence of multicollinearity, homogeneity of variances, normality of residuals and random effects, singularity) were checked using the package *performance* [57] and *nlme* [56] and were adequately met. The detailed script and results can be obtained from the RMarkdown output file in the supplementary material. Lastly, preregistered Pearson's and Spearman's correlations were computed between changes in the subscales of the FCQ and *cortisol* as well as *glucose reactivity*.

In addition to the above described preregistered confirmatory and exploratory analyses, we performed two sensitivity analyses. First, as the single imputation approach for missing values may bias the results, we implemented predictive mean matching (PMM) on item level using the *mice* package [58] to generate ten imputed data files, which were merged using the function *merge_imputations* (.) from the *sjmisc* package before rerunning the analysis [59]. Second, we rerun all linear mixed models using commonly used covariates, i.e., sex, age and BMI.

### 2.6 Power analysis

Based on our design matrix (2 × 2 design with two repeated measures of the FCQ state), a sensitivity power analysis indicated that with a repeated measures ANOVA, we have 80 % power to detect a small to medium interaction effect sizes of at least *f* = .217 at α = .05 (assuming a correlation between repeated measures of *r* = .5 and non-sphericity correction of ε = 1).

## 3 Results

### 3.1 Descriptives and preliminary analyses

On average, participants fasted for approximately 6 h (*SD* = 2.9) and had a blood glucose concentration of 103.41 mg/dL upon arrival (*SD* = 11.43; range: 66-127). The four groups neither differed in *food craving* upon arrival, *F* (3, 58) = 1.83, *p* = .152, _part_eta^2^ = .09, nor in *stress eating tendencies*, *F* (3, 58) = .48, *p* = .697, _part_eta^2^ = .02. Descriptives of the sample are provided in Table 1. The four groups differed significantly in how long they fasted upon arrival (see description of *time since the last meal* in Table 1). Fasting duration was neither significantly associated with *food craving* before or after the TSST nor with *changes in food craving*.

**Table 1:** Description of the sample.

	*control water (n = 14)*	*control sugar (n = 16)*	*stress water (n = 18)*	*stress sugar (n = 14)*	*complete sample (N = 62)*	*p value*
age (in years)	23.57 (4.31)	22.81 (2.01)	23.06 (2.62)	23.14 (3.18)	23.13 (3.02)	*p* = .925
sex (number of females [%])	7 (50 %)	8 (50 %)	11 (61 %)	8 (57 %)	34 (55 %)	*p* = .897
Body mass index (in kg/m^2^)	23.60 (2.27)	22.60 (3.25)	21.74 (2.01)	22.47 (3.00)	22.55 (2.68)	*p* = .287
Blood glucose baseline (in mg/dL)	103.05 (9.24)	106.05 (12.46)	103.56 (8.55)	99.62 (15.03)	103.41 (11.43)	*p* = .446
Cortisol baseline (in nmol/L)	5.36 (2.90)	4.05 (2.75)	4.72 (5.41)	5.29 (3.96)	4.82 (3.93)	*p* = .788
Food craving (pre TSST)	37.34 (9.49)	37.13 (13.02)	45.00 (10.91)	42.14 (12.10)	40.59 (11.71)	*p* = .152
Food craving (post TSST)	47.64 (11.96)	38.25 (14.28)	51.38 (14.54)	39.00 (12.49)	44.35 (14.36)	***p* = .016**
Stress eating tendency	2.91 (.65)	2.82 (.55)	2.79 (.79)	2.61 (.67)	2.79 (.67)	*p* = .697
Time since last meal (in hours)	8.11 (4.54)	5.65 (.91)	5.21 (.51)	7.17 (3.43)^a^	6.42 (2.90)	***p* = .017**
Chronic stress (PSS sum score)	25.57 (4.54)	23.75 (6.65)	26.61 (6.77)	23.14 (4.80)	24.85 (5.91)	*p* = .323

*Note.* If not otherwise specified, values represent mean (*SD*). P-values indicate significant differences in the listed variables as obtained from comparisons of the experimental groups using ANOVAs or Chi-squared tests. TSST = Trier Social Stress Test. PSS = Perceived Stress Scale. ^a^: *n* = 1 missing.

We tested whether any of the potential covariates were significantly correlated with *changes in food craving* and found that *stress eating tendency* was associated with a significant increase in food craving over time, *r* (60) = .27, *p* = .032. This association was significant in the group exposed to the friendly control condition, *r* (28) = .44, *p* = .016, but not the stress condition, *r* (30) = .14, *p* = .445. *Stress eating tendency* did not differ between men and women, *F* (1, 60) = 2.14, *p* = .149, _part_eta^2^ = .03. Based on these results, we decided to control for the interaction term *stress eating tendency* x *time* in the subsequent confirmatory analyses. The Pearson's correlation matrix depicting the coefficients of all potential covariates is provided in the supplemental material (Figure S1).

### 3.2 Manipulation checks

The success of the stress and glucose manipulations was confirmed in a previous publication on this study [32]. In short, the stressful TSST led to a significantly stronger cortisol and subjective stress response as compared with the friendly control TSST (cf. Supplementary Fig. S2). Of note, cortisol stress reactivity was weaker as compared with previous TSST studies comprising both the speech as well as the mental arithmetic task and the friendly control TSST was subjectively arousing. Blood glucose levels rose significantly after *glucose* but not *water* consumption, with peak levels being reached approximately 60 min after *glucose* consumption (mean third glucose measurement after *glucose* drink = 180.45 mg/dL; *water* drink = 99.93 mg/dL). Previous analyses also showed that participants consuming *glucose* liked the drink significantly less, rated the taste as significantly sweeter and had a lower desire to drink more as compared with those who consumed *water* [32].

### 3.3 Effect of stress on changes in food craving

The linear mixed model predicting food craving over *time* by *stress* condition and *drink* condition included 124 observations nested in 62 participants (marginal *R*^2^ = .060; conditional *R*^2^ = .905). We included a random intercept for participants, random slope for *time*, and a linear effect of *time* before adding the effects of interest of each hypothesis. To test H1a, we added the main and interaction effects of *stress* or *cortisol reactivity*.

We found a significant main effect of *time, beta* = .41, *SE* = .13, *p* = .004, indicating a significant increase in food craving across the experiment. Further, we found a main effect of *stress*, *beta* = .49, *SE* = .22, *p* = .027, indicating higher average food craving in the stress as compared to the friendly control condition. The interaction between *stress* and *time* was not significant (see Supplementary Material Table S2). Exploratory post hoc *t*-tests indicated that food craving increased significantly in response to the friendly control TSST (*p* = .004), but not to the stressful TSST (*p* = .203).

**Fig. 2:**
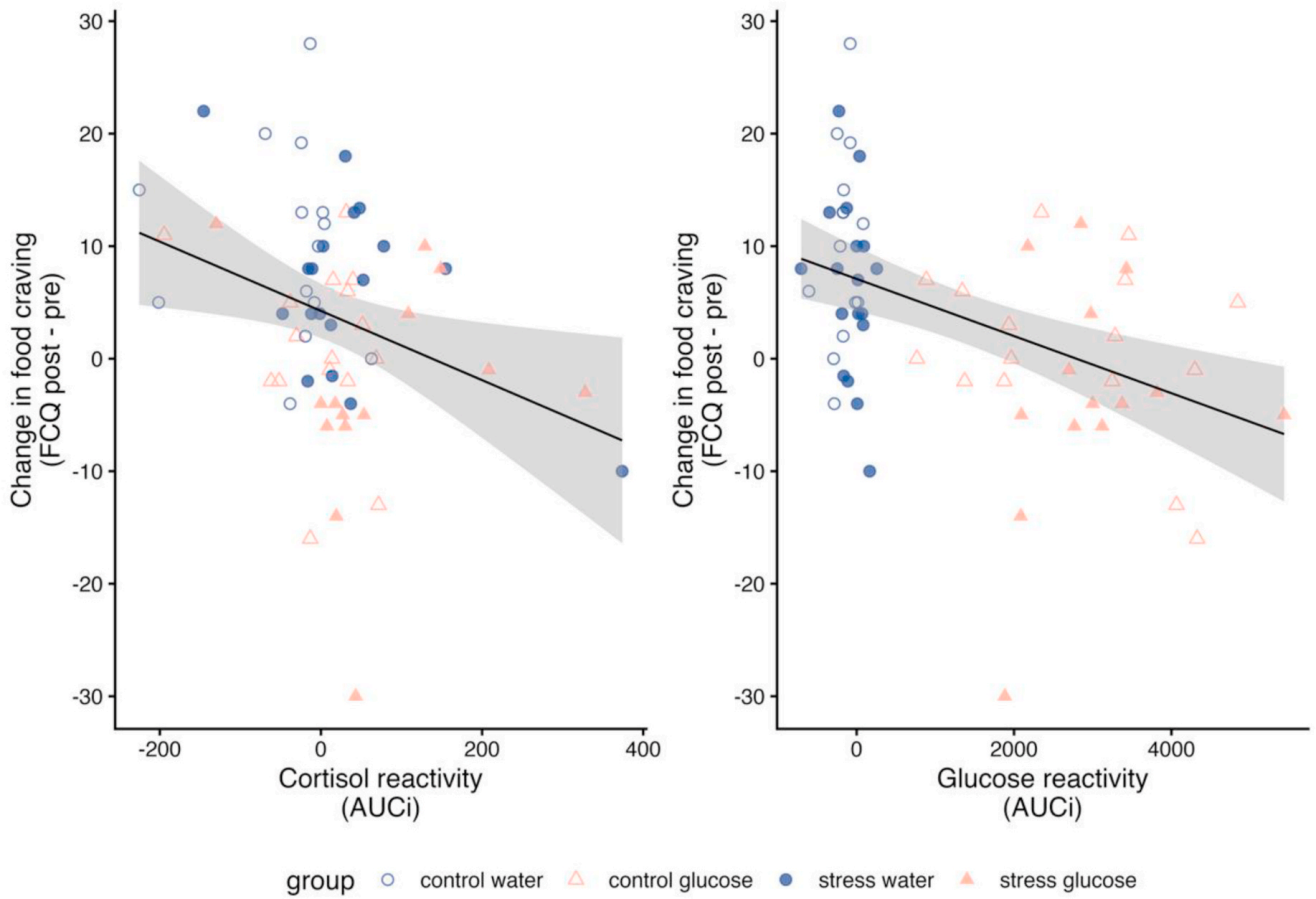
Scatterplots depicting the association between cortisol reactivity (left panel) and glucose reactivity (right panel) and changes in food craving over time in the four experimental conditions. A positive change in food craving represents a relative increase in craving over time, while a negative change in food craving represents a relative decrease in craving over time.

When using *cortisol reactivity* as a predictor (marginal R^2^ = .040; conditional R^2^ = .904), we could confirm the significant change in food craving over *time*, *beta* = .29, *SE* = .09, *p* = .003. In addition, we found a significant *time* x *cortisol reactivity* interaction, *beta* = −.23, *SE* = .09, *p* = .015, indicating that a higher *cortisol reactivity* was associated with a decrease in *food craving* whereas a lower *cortisol reactivity* was associated with an increase in *food craving* from pre to post TSST (see Supplementary Table S3). For descriptive purposes, this relationship is depicted using *change in food craving* as outcome variable in Fig. 2 (left panel).

Adding the *stress eating tendency* × *time* interaction term to the models did not change the interpretation of the results, but additionally revealed a significant *stress eating tendency* × *time* interaction effect (stress condition model: *beta* = .19, *SE* = .09, *p* = .043; stress reactivity model: *beta* = .19, *SE* = .09, *p* = .039), indicating that those with a higher *stress eating tendency* also exhibited higher post-stress food craving (see Supplementary Tables S4 and S5).

### 3.4 Effect of stress and drink on changes in food craving

**Fig. 3:**
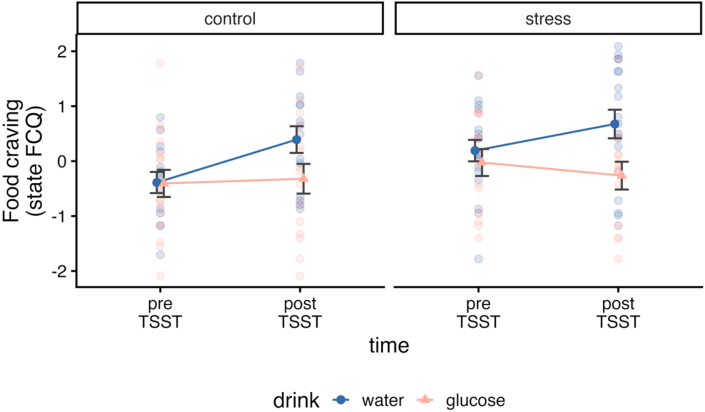
Changes in food craving over time. State FCQ = Food Craving Questionnaire (state version total score). TSST = Trier Social Stress Test.

The model in which we predicted food craving from *stress* condition and *drink* condition (marginal *R*^*2*^ = .151; conditional *R*^*2*^ = .917) indicated a significant main effect of *time*, *beta* = .78, *SE* = .18, *p* < .001. The main effect of *stress* did not remain significant, *beta* = .58, *SE* = .31, *p* = .066, but we found a significant *time* × *drink* interaction effect, *beta* = −.70, *SE* = .24, *p* = .005. Post hoc *t*-tests indicated that food craving increased significantly from before to after the TSST in the groups that consumed *water* (friendly control, water: *p* < .001; stress, water: *p* = .003), but not in the groups that consumed *glucose* (friendly control, glucose: *p* = .605; stress, glucose: *p* = .180). No other main or interaction effects reached statistical significance (see Supplementary Table S6). Adding the *stress eating tendency* × *time* interaction term to the models did not change the interpretation of the results (see Supplementary Table S7). Changes in food craving over time are depicted in Fig. 3.

When using *glucose reactivity* and *cortisol reactivity* as predictors (marginal *R*^*2*^ = .101; conditional *R*^*2*^ = .914), we again confirmed the main effect of *time*, *beta* = .27, *SE* = .08, *p* = .002, as well as a significant *time* x *cortisol reactivity* interaction, *beta* = −.18, *SE* = .09, *p* = .037. In addition, we found a significant *time* x *glucose reactivity* interaction, *beta* = −.29, *SE* = .09, *p* < .001, indicating that a higher *glucose reactivity* was associated with a relative decrease in *food craving* over *time* (see Supplementary Table S8). For descriptive purposes, this relationship is depicted using *change in food craving* as outcome variable in Fig. 2 (right panel). No other interaction effects reached statistical significance.

Adding the *stress eating tendency* × *time* interaction term to the model did not change the interpretation of the results but revealed a significant *stress eating tendency* × *time* interaction effect, *beta* = .17, *SE* = .08, *p* = .047, indicating that a higher *stress eating tendency* was associated with higher post-TSST food craving (see Supplementary Table S9).

### 3.5 Exploration of sex-specific effects

To explore whether *sex* modulates the effect of glucose on stress-induced changes in food craving, we preregistered to add the main and interaction effects of *sex* to the mixed-effects models described above. However, adding the effects did not result in significant increases in model fits (*stress* and *drink* condition model: main effect *p* = .556, interaction effect *p* = .064; *cortisol reactivity* and *glucose reactivity* model: main effect *p* = .847; interaction effect *p* = .193). For interested readers, the coefficients obtained from the evaluation of the models are provided in the supplementary material (Tables S10 and S11). In light of the low numbers of males and females in each group (cf. Table 1), the statistical power to detect a potential sex modulation was extremely compromised in the current dataset.

### 3.6 Correlational analyses

**Fig. 4:**
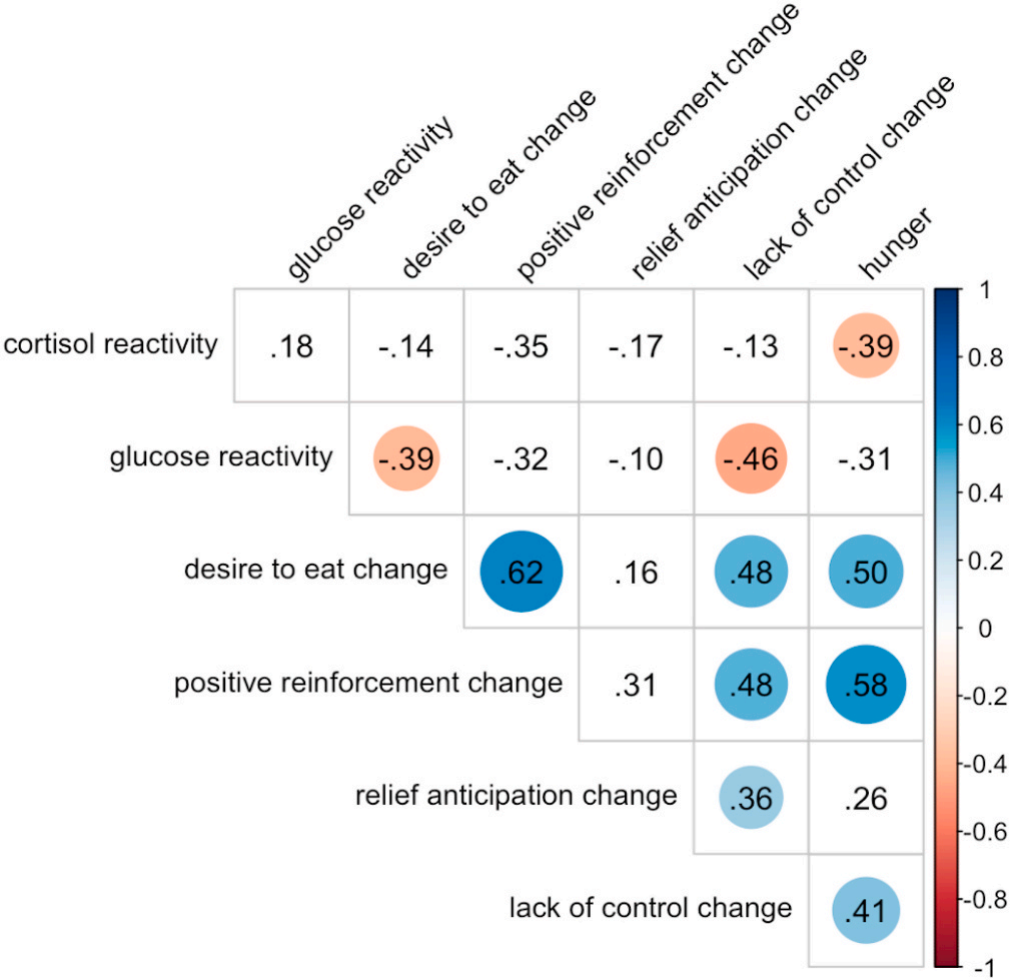
Correlation matrix depicting Pearson's correlation coefficients between cortisol and glucose reactivity and changes in facets of food craving. Statistically significant coefficients that survive a Bonferroni correction for 11 tests (α _adj_ = .005) are shaded with a colored circle (blue = positive association, red = negative association).

A matrix depicting Pearson's correlation coefficients between *cortisol reactivity, glucose reactivity* as well as *changes in food craving* aspects, i.e., changes in desire to eat, positive reinforcement, relief anticipation, lack of control over eating, and physiological hunger from pre to post TSST, is depicted in Fig. 4. Correlations surviving a Bonferroni correction for 11 tests (correlation of five subscales of the FCQ with cortisol and glucose reactivity as well as correlation between cortisol and glucose reactivity, α _adj_ = .005) are marked with a circle in the Figure and an asterisk in the text. Higher *cortisol reactivity* was significantly correlated with a decrease in the expected positive reinforcement from eating, *r* = −.35, *p* = .007, and a decrease in hunger, *r* = −.39, *p* = .002*. Higher *glucose reactivity* was significantly associated with a decrease in the desire to eat, *r* = −.39, *p* = .002*, positive reinforcement, *r* = −.32, *p* = .013, lack of control, *r* = −.46, *p* < .001*, and hunger, *r* = −.31, *p* = .014. The same pattern emerged when using Spearman's correlation coefficients (see Supplementary Material Fig. S3). Additionally, there was a significant monotonic (yet not linear) relationship between *cortisol reactivity* and *glucose reactivity*, Spearman's correlation coefficient, *r* = .30, *p* = .016, which did not survive Bonferroni correction. Further, a Spearman correlation between residualized cortisol and glucose reactivity (controlling for drink and condition) was not significant, *r* = .07, *p* = .577, suggesting that the monotonic association between cortisol and glucose reactivity may be largely explained by the experimental manipulation (drink and/or condition), rather than a general relationship between the two physiological responses.

### 3.7 Sensitivity analyses (not preregistered)

We reran all analyses with a dataset in which missing values in blood glucose, cortisol and the FCQ were imputed by *Predictive Mean Matching* instead of using a single imputation approach. Implementing this approach did not change the interpretation of the results. The detailed script and results can be obtained from the RMarkdown output file in the supplementary material.

In addition, we reran all linear mixed models while controlling for *sex*, *age* and *BMI*, as well as their interaction with *time*. In most models (H1a, H1b, H2a), we could replicate the significance of the previously observed effects. Yet, the effects of the model in which we predicted changes in *food craving* over *time* by *cortisol* and *glucose reactivity* could be replicated only partially (H2b): When controlling for *sex*, *age* and *BMI*, we found a significant main effect of *time*, *beta* = .39, *SE* = .11, *p* = .001, a significant *glucose reactivity × time* interaction, *beta* = −.29, *SE* = .08, *p* = .001, but no evidence for a *cortisol reactivity × time* interaction, *beta* = −.15, *SE* = .09, *p* = .087 (marginal *R*^*2*^ = .161; conditional *R*^*2*^ = .92). Of note, the beta coefficient still implied a negative association between *cortisol reactivity* and changes in *food craving* over *time*. The detailed results can be obtained from the supplementary material (cf. Tables S12–S15).

## 4 Discussion

The present analysis examined the effect of glucose and stress on changes in food craving. Contrary to our hypothesis, a greater increase in cortisol was associated with a relative decrease in food craving, more specifically, a reduction in physiological hunger. A glucose preload and the associated rise in blood glucose concentrations led to reductions in food craving, specifically to a decrease in the desire to eat and a decrease in the lack of control over eating. When descriptively comparing the effect sizes, *glucose reactivity* (*beta = *−.29) was a stronger predictor for changes in food craving than *cortisol reactivity* (*beta = *−.18). However, the interpretation of this finding needs careful consideration of the high glucose dose (75 g) that was administered and may have confounded the results by inducing satiety, and the modest cortisol stress reactivity observed in the current setting. We found no support for the notion that glucose and cortisol reactivity interactively affected craving, suggesting that these processes may operate via partially distinct pathways. Given limited statistical power, this null result should be interpreted with caution and replication attempts incorporating a stronger stressor with a clearer differentiation from the control condition, as well as a larger sample should be awaited before drawing final conclusions. To summarize, these findings provide preliminary evidence that – in the current setting – the glucose manipulation was more strongly associated with food craving following acute stress than cortisol responses, and that acute cortisol elevations may not universally promote food craving.

Based on reported effects of cortisol on hunger signaling and the reward system, we expected that participants exposed to stress would exhibit a greater increase in food craving as compared to those in the friendly control condition. Contrary to this hypothesis, food craving significantly increased in the control but not the stress group and higher cortisol reactivity was associated with reductions in food craving. These findings contrast with studies reporting that higher cortisol reactivity is linked to greater food intake (e.g. [2,22]) but align with more recent work demonstrating a negative relationship between cortisol reactivity and self-reported hunger in a healthy sample [24]. This negative association between acute stress and food craving is also congruent with findings from animal models in which acute stress has been linked to reduced food intake [[60], [61], [62]]. In these studies, metabolic mediators including ghrelin [62] and GLP-1 [61] have been associated with the change in food intake, which may be worth exploring in the future. The reductions in hunger may reflect an adaptive response in which the organism prioritizes fight-or-flight processes and temporarily suppresses non-essential functions, including food intake [63]. While thereby, stress may result in attenuated food motivation in healthy, non-restrained eaters (e.g. [64]), this mechanisms may be disturbed in vulnerable populations, i.e. in individuals with obesity, chronic stress, or disordered eating behavior, which have often been reported to increase food intake following stress [23,[65], [66], [67]]. Likewise, chronic stress has also been associated with increased food intake of palatable food in animals, which is discussed to be mediated by dopaminergic pathways [68]. In the current study, chronic stress was however not associated with changes in food craving. As cortisol regulates motivating behavior [69] and elevated cortisol stress reactivity increases the reliance on habits at the expense of goal-directed behavior [64], these factors may offer potential target points for future work that compares predictors of stress-induced changes in eating behavior and food intake more systematically in healthy and at-risk populations. At the same time, it needs to be acknowledged that we found a negative link between cortisol reactivity and food craving rated approximately 35 min after stressor onset. Thus, the average peak cortisol levels had already been reached, and arousal had already faded (cf. Supplementary Fig. S2), suggesting that we captured delayed effects of stress on food craving in our study. Future work should aim to elucidate whether the link between cortisol stress reactivity and food craving dynamically changes across the post-stress period.

Besides the effect of cortisol reactivity, we observed that the high sugary preload and the associated strong increase in blood glucose concentrations strongly reduced food craving. This finding aligns with previous work showing that sugar consumption generally increases satiety and reduces subsequent food intake [70]. In normal-weight women, a glucose vs. water infusion to the stomach did not impact food craving, despite rising blood glucose concentrations and changing brain activation to food cues [28]. Similarly, in women with obesity, no difference in food craving after glucose vs. water infusion to the stomach was found [28]. At the same time, no evidence for a significant difference in food intake following a preload with either sugar or non-caloric sweeteners was observed, indicating that sweet taste alone may act as a strong signal in this context [70,71]. This suggests that an active ingestion of glucose, and the perception of its sweet taste, may have driven the reductions in food craving in our study, potentially by influencing food-related cognitions. As the effect of glucose on changes in food craving were rather strong, including a sugary preload in experimental studies that assess changes in food craving in stressful and non-stressful contexts should be considered cautiously, as metabolic effects may mask those of other manipulations that exhibit more subtle influences. While glucose dynamics were associated with food craving, they did not completely mask the effects of stress in our sample. Cortisol reactivity was negatively associated with craving, although stress responses were only modest compared with the classic TSST paradigm [72] and the control condition was subjectively arousing. The fact that the effects of cortisol reactivity on food craving were not completely absent highlights that both metabolic-as well as stress-related factors are potent modulators of food craving.

Contrary to our hypothesis, we did not find evidence for an interaction between *glucose* and *cortisol reactivity* on food craving. Given the fact that both the weak cortisol stress response to the TSST and the increase in arousal in response to the friendly TSST may have reduced the differentiation between conditions, as well as a lack of statistical power to detect interaction effects, it is possible that the current setting provoked an underestimation of the stress effect which should be followed up upon in future research. At the same time, a monotonic –yet not linear– relation between glucose reactivity and cortisol reactivity was observed: Those with a higher increase in blood glucose levels also tended to exhibit a higher cortisol stress response. Importantly, this effect did not survive the correction for multiple testing and should be interpreted with caution. In the past, a positive link between glucose and cortisol levels has been reported in some [44,73], but not all previous studies [46,74,75]. Of note, throughout all analyses, relations between glucose preload, stress, and changes in food craving were stronger when analyzing interindividual differences in cortisol as well as glucose reactivity as compared with average group effects, which suggest that assessing fine-grained interindividual differences may be pivotal to better understand which factors shape food craving in stressful and non-stressful contexts. Thereby, potential modulations by both trait (e.g., stress eating tendency, stress coping) as well as state variables (e.g., elicited emotions, availability of food) should also be considered.

Previous research suggested that sex plays a moderating role in stress-induced eating and food craving. For example, women tend to exhibit higher levels of emotional food craving, whereas men are more likely to score higher on reward-driven or outcome-focused craving dimensions [76]. Additionally, women are more prone to stress-induced eating for emotional or hedonic purposes, such as mood regulation or comfort [2,77], and hormonal fluctuations across the menstrual cycle have been shown to influence appetite and eating behavior in women [78]. In our sample, we found that self-rated tendencies to increase food intake when stressed was linked to higher post-TSST food craving. Yet we neither confirmed higher tendencies to change eating behavior in response to stress in women as compared with men, nor could we observe that stress-induced changes in food craving were modulated by sex. While our sample size may have been too small to detect the hypothesized effects, future work should study the effects of sex on stress-induced eating and food craving more systematically.

Our study has several strengths, including the manipulation of blood glucose levels prior to stress, the use of a well-established stress paradigm and a friendly control condition, the inclusion of physiological markers that confirmed the success of our experimental manipulations, and the observation of individual changes in food craving in direct proximity of the stressor. Nevertheless, some limitations should be mentioned when interpreting our findings. First, the cortisol increases in this study that were evoked through the TSST stress condition were relatively weak compared with other studies [72]. In contrast, the glucose manipulation was rather strong. This may have attenuated observable stress effects, which is particularly relevant to the finding that glucose was a stronger predictor of food craving than cortisol and the lack of evidence of an interaction between both factors. Second, participants could infer from the sweet taste of the glucose drink whether they were allocated to the glucose or water condition, thereby comprising blinding upon drink ingestion. Also, participants liked the glucose drink significantly less and had a lower desire to drink more as compared with those who consumed water [32]. This may have critically biased the self-reported craving ratings, which are susceptible to expectancy effects [79], which reduces the internal validity of our approach. To disentangle the role of expectations formed by sweet taste from actual metabolic effects, future studies should include a control condition with a non-caloric sweetened drink. This would further help to clarify whether the observed effects are driven by physiological energy availability or by the hedonic perception of sweetness. In addition, our pre–post design captured craving levels at baseline and approximately 20–30 min after the end of the stressor, at the expected cortisol peak. Assessments at multiple time points would have yielded a more nuanced understanding of stress-related changes in food motivation. Furthermore, we assessed subjective craving by self-report, which may not directly translate to actual food intake. Of note, while the state version of the FCQ questionnaire reliably captures momentary changes in food craving [53], we did not find an association between stress eating tendency and an increase in self-reported food craving post stressor exposure. The inclusion of objective measures of food intake across different food categories, or the use of food-cue paradigms (e.g., food picture presentation), would have provided additional insight into behavioral outcomes and self-control capacity under stress. Importantly, a meta-analysis by Hill et al. [80] demonstrated that stress is associated with increased consumption of unhealthy foods and decreased consumption of healthy foods, highlighting a clinically relevant distinction that may not be fully captured by self-reported craving measures alone. In addition, the generalizability and ecological validity of the study is limited due to the young sample (mean age = 23.13) and the long fasting window (5 h) before the session which may also not adequately represent real-life eating behavior. Lastly, while this study focused primarily on biological mechanisms, it only partially accounted for individual psychological factors that may influence stress-induced food craving, such as dietary restraint [11], which has been associated with a reduced sensitivity to internal hunger and satiety cues that may be amplified under stress [81]. Future work would benefit from integrating additional validated measures of eating behavior styles to better capture the complex interplay between hormonal, behavioral, and sociocultural factors that may influence food craving.

Taken together, the present findings contribute to a growing body of research indicating that stress-related changes in food craving are not uniformly driven by cortisol reactivity, but instead reflect a complex interplay of psychological, metabolic, and hormonal factors. Our work did not provide evidence for an interaction between glucose and cortisol, which suggests that metabolic and stress-related processes may at least partially contribute independently to short-term fluctuations in food craving. Future research should replicate these findings in larger, more diverse samples with increased statistical power that ideally allow to evaluate sex- and menstrual-cycle phase-specific effects. Incorporating longitudinal designs could clarify whether the observed short-term reductions in post-stress craving translate into meaningful changes in eating behavior on the long run or when exposed to stressors repeatedly. This will be crucial to refining our understanding of when, how, and for whom acute stress influences appetite regulation and eating behavior, to advancing theoretical models of stress-induced eating, and to defining risk factors and ultimately informing targeted interventions for vulnerable populations.

## Supplementary information

Supplementary information, data and the analysis code can be retrieved from the following Open Science Framework (OSF) project: https://osf.io/5wt8r/overview.

## Declaration of generative AI and AI-assisted technologies in the manuscript preparation process

During the preparation of this work the author(s) used ChatGPT for improving language and readability as well as Consensus. app for a scoping search of additional literature beyond the classical search on literature databases. After using the tools, we reviewed and edited the content as needed and take full responsibility for the content of the published article.

## Funding

This work was funded by a Young Scholar Fund awarded to Maria Meier by the Committee of Research of the University of Konstanz.

## CRediT authorship contribution statement

**Flora Nasilowski:** Conceptualization, Data curation, Formal analysis, Investigation, Writing – original draft. **Julia Reichenberger:** Writing – review & editing. **Bernadette Denk:** Writing – review & editing. **Raphaela Gaertner:** Writing – review & editing. **Elea Klink:** Writing – review & editing. **Eva Unternaehrer:** Formal analysis, Validation, Writing – review & editing. **Nina Volkmer:** Writing – review & editing. **Stella Wienhold:** Writing – review & editing. **Jens Pruessner:** Conceptualization, Methodology, Resources, Supervision, Writing – review & editing. **Maria Meier:** Conceptualization, Data curation, Formal analysis, Funding acquisition, Investigation, Methodology, Project administration, Supervision, Visualization, Writing – original draft.

## Declaration of competing interest

None.
