# Supplementary material for: The influence of glucose intake and psychosocial stress on food craving in healthy adults
Source: Compr Psychoneuroendocrinol. 2026 Aug 5;27:100372. doi: 10.1016/j.cpnec.2026.100372 (PMC13487594; doi:10.1016/j.cpnec.2026.100372)
Supplement: Supplementary file 1 [file mmc1.docx]

***Supplementary material for* The influence of glucose intake and psychosocial stress on food craving in healthy adults**

Flora Nasilowski^1^, Julia Reichenberger^2^, Bernadette Denk^1,3^, Raphaela Gaertner^1^, Elea Klink^1^, Eva Unternaehrer^4^, Nina Volkmer^1,3^, Stella Wienhold^1,3^, Jens Pruessner^1,3^, Maria Meier^1, 5^

1 *Department of Neuropsychology, University of Konstanz, Konstanz, Germany*
2 *Department of Psychology, Centre for Cognitive Neuroscience, Paris-Lodron-University of Salzburg, Salzburg, Austria*
3 *Centre for the Advanced Study of Collective Behavior, Konstanz, Germany*
4 *University Clinics Basel (UPK), University of Basel, Basel, Switzerland*
5 *University of Ulm, Germany*

Correspondence: Maria Meier ([maria.meier@uni-konstanz.de](mailto:maria.meier@uni-konstanz.de))

***Table S1.*** Model formula of the linear mixed models testing the hypotheses outlined in the main text.

| *Hypothesis* | *Fixed effects* | *Random effects* |
| --- | --- | --- |
| *Covariate unadjusted models (preregistered)* | | |
| H1a – unadjusted | food_craving ∼ time + condition + time:condition | (time ∣ ID) |
| H1b – unadjusted | food_craving ∼ time + AUCi_cort + time:AUCi_cort | (time ∣ ID) |
| H2a – unadjusted | food_craving ~ time + condition + drink + time:condition + time:drink + condition:drink + time:condition:drink | (time ∣ ID) |
| H2b – unadjusted | food_craving ~ time + AUCi_cort + AUCi_glucs + time: AUCi_cort + time:AUCi_glucs + AUCi_cort:AUCi_glucs + time:AUCi_cort:AUCi_glucs | (time ∣ ID) |
| *Adjusted models: Covarying for stress eating tendency (SSES) x time interaction (preregistered)* | | |
| H1a – adjusted | food_craving ~ time + condition + SSES + time:condition + time:SSES | (time ∣ ID) |
| H1b – adjusted | food_craving ~ time + AUCi_cort + SSES + time: AUCi_cort + time:SSES | (time ∣ ID) |
| H2a – adjusted | food_craving ~ time + condition + drink + SSES + time:condition + time:drink + condition:drink + time:SSES + time:condition:drink | (time ∣ ID) |
| H2b – adjusted | food_craving ~ time + AUCi_cort + AUCi_glucs + SSES + time:AUCi_cort + time:AUCi_glucs + AUCi_cort:AUCi_glucs + time:SSES + time:AUCi_cort:AUCi_glucs | (time ∣ ID) |
| *Exploration: Covarying for sex x time interaction (preregistered)* | | |
| H1a – adjusted | food_craving ~ time + condition + sex + time:condition + time: sex | (time ∣ ID) |
| H1b – adjusted | food_craving ~ time + AUCi_cort + sex + time: AUCi_cort + time: sex | (time ∣ ID) |
| H2a – adjusted | food_craving ~ time + condition + drink + sex + time:condition + time:drink + condition:drink + time: sex + time:condition:drink | (time ∣ ID) |
| H2b – adjusted | food_craving ~ time + AUCi_cort + AUCi_glucs + sex + time:AUCi_cort + time:AUCi_glucs + AUCi_cort:AUCi_glucs + time: sex + time:AUCi_cort:AUCi_glucs | (time ∣ ID) |
| *Sensitivity analysis: Covarying for sex, age and BMI and their interaction with time (not preregistered)* | | |
| H1a – adjusted | food_craving ~ time + condition + age + sex + BMI + time:condition + time:age + time:sex + time:BMI | (time ∣ ID) |
| H1b – adjusted | food_craving ~ time + AUCi_cort + age + sex + BMI + time:AUCi_cort + time:age + time:sex + time:BMI | (time ∣ ID) |
| H2a – adjusted | food_craving ~ time + condition + drink + age + sex + BMI + time:condition + time:drink + condition:drink + time:age + time:sex + time:BMI + time:condition:drink | (time ∣ ID) |
| H2b – adjusted | food_craving ~ time + AUCi_cort + AUCi_glucs + age + sex + BMI + time:AUCi_cort + time:AUCi_glucs + AUCi_cort:AUCi_glucs + time:age + time:sex + time:BMI + time: AUCi_cort:AUCi_glucs | (time ∣ ID) |
| *Note.* “:” refers to an interaction effect. Time = pre vs. post TSST. Condition = friendly vs. stressful TSST. Drink = water vs. glucose. BMI = Body Mass Index. SSES = Salzburger Stress Eating Scale. ID = Participant identifier. | | |

***Figure S1.*** Pearson’s correlation matrix between potential covariates and changes in food craving (FCQ total change). Statistically significant coefficients are shaded with a colored circle (blue = positive association, red = negative association).

***Figure S2.*** Subjective stress ratings (panel A) and salivary cortisol (panel B) over the course of the experiment.

***Table S2.*** Evaluation of the model in which we predicted Food Craving by time (2 levels: pre, post) and condition (2 levels: stress, control).

|  | **value** | | |
| --- | --- | --- | --- |
| *Predictors* | *Estimates* | *std. Error* | *p* |
| (Intercept) | -0.40 | 0.16 | **0.014** |
| variable [FCQ_total_post] | 0.41 | 0.13 | **0.004** |
| condition [stress] | 0.49 | 0.22 | **0.027** |
| variable [FCQ_total_post] × condition [stress] | -0.24 | 0.19 | 0.202 |
| **Random Effects** | | | |
| σ^2^ | 0.09 | | |
| τ_00_ _code_ | 0.62 | | |
| τ_11_ _code.variableFCQ_total_post_ | 0.34 | | |
| ρ_01_ _code_ | 0.11 | | |
| ICC | 0.90 | | |
| N _code_ | 62 | | |
| Observations | 124 | | |
| Marginal R^2^ / Conditional R^2^ | 0.060 / 0.905 | | |

***Table S3.*** Evaluation of the model in which we predicted Food Craving by cortisol reactity (AUCi) and time (2 levels: pre, post).

|  | **value** | | |
| --- | --- | --- | --- |
| *Predictors* | *Estimates* | *std. Error* | *p* |
| (Intercept) | -0.14 | 0.11 | 0.215 |
| variable [FCQ_total_post] | 0.29 | 0.09 | **0.003** |
| AUCi cort | 0.03 | 0.11 | 0.779 |
| variable [FCQ_total_post] × AUCi cort | -0.23 | 0.09 | **0.015** |
| **Random Effects** | | | |
| σ^2^ | 0.10 | | |
| τ_00_ _code_ | 0.68 | | |
| τ_11_ _code.variableFCQ_total_post_ | 0.30 | | |
| ρ_01_ _code_ | 0.06 | | |
| ICC | 0.90 | | |
| N _code_ | 62 | | |
| Observations | 124 | | |
| Marginal R^2^ / Conditional R^2^ | 0.040 / 0.904 | | |

***Table S4.*** Evaluation of the model in which we predicted Food Craving by time (2 levels: pre, post) and condition (2 levels: stress, control) while controlling for stress eating tendency (SSES).

|  | **value** | | |
| --- | --- | --- | --- |
| *Predictors* | *Estimates* | *std. Error* | *p* |
| (Intercept) | -0.41 | 0.16 | **0.011** |
| variable [FCQ_total_post] | 0.39 | 0.13 | **0.005** |
| condition [stress] | 0.52 | 0.22 | **0.020** |
| SSES mean | 0.12 | 0.11 | 0.264 |
| variable [FCQ_total_post] × condition [stress] | -0.20 | 0.18 | 0.284 |
| variable [FCQ_total_post] × SSES mean | 0.19 | 0.09 | **0.043** |
| **Random Effects** | | | |
| σ^2^ | 0.09 | | |
| τ_00_ _code_ | 0.61 | | |
| τ_11_ _code.variableFCQ_total_post_ | 0.31 | | |
| ρ_01_ _code_ | 0.05 | | |
| ICC | 0.90 | | |
| N _code_ | 62 | | |
| Observations | 124 | | |
| Marginal R^2^ / Conditional R^2^ | 0.117 / 0.908 | | |

***Table S5.*** Evaluation of the model in which we predicted Food Craving by cortisol reactity (AUCi) and time (2 levels: pre, post) while controlling for stress eating tendency (SSES).

|  | **value** | | |
| --- | --- | --- | --- |
| *Predictors* | *Estimates* | *std. Error* | *p* |
| (Intercept) | -0.14 | 0.11 | 0.216 |
| variable [FCQ_total_post] | 0.29 | 0.09 | **0.002** |
| AUCi cort | 0.04 | 0.11 | 0.734 |
| SSES mean | 0.10 | 0.11 | 0.401 |
| variable [FCQ_total_post] × AUCi cort | -0.21 | 0.09 | **0.019** |
| variable [FCQ_total_post] × SSES mean | 0.19 | 0.09 | **0.039** |
| **Random Effects** | | | |
| σ^2^ | 0.09 | | |
| τ_00_ _code_ | 0.67 | | |
| τ_11_ _code.variableFCQ_total_post_ | 0.27 | | |
| ρ_01_ _code_ | 0.01 | | |
| ICC | 0.90 | | |
| N _code_ | 62 | | |
| Observations | 124 | | |
| Marginal R^2^ / Conditional R^2^ | 0.085 / 0.907 | | |

***Table S6.*** Evaluation of the model in which we predicted Food Craving by condition (2 levels: stress, control), drink (2 levels: glucose, water) and time (2 levels: pre, post).

|  | **value** | | |
| --- | --- | --- | --- |
| *Predictors* | *Estimates* | *std. Error* | *p* |
| (Intercept) | -0.39 | 0.23 | 0.100 |
| variable [FCQ_total_post] | 0.78 | 0.18 | **<0.001** |
| condition [stress] | 0.58 | 0.31 | 0.066 |
| drink [sugar] | -0.02 | 0.32 | 0.959 |
| variable [FCQ_total_post] × condition [stress] | -0.30 | 0.23 | 0.209 |
| variable [FCQ_total_post] × drink [sugar] | -0.70 | 0.24 | **0.005** |
| condition [stress] × drink [sugar] | -0.20 | 0.44 | 0.654 |
| (variable [FCQ_total_post] × condition [stress]) × drink [sugar] | -0.03 | 0.34 | 0.937 |
| **Random Effects** | | | |
| σ^2^ | 0.08 | | |
| τ_00_ _code_ | 0.63 | | |
| τ_11_ _code.variableFCQ_total_post_ | 0.24 | | |
| ρ_01_ _code_ | 0.04 | | |
| ICC | 0.90 | | |
| N _code_ | 62 | | |
| Observations | 124 | | |
| Marginal R^2^ / Conditional R^2^ | 0.151 / 0.917 | | |

***Table S7.*** Evaluation of the model in which we predicted Food Craving by condition (2 levels: stress, control), drink (2 levels: glucose, water) and time (2 levels: pre, post) while controlling for stress eating tendency (SSES).

|  | **value** | | |
| --- | --- | --- | --- |
| *Predictors* | *Estimates* | *std. Error* | *p* |
| (Intercept) | -0.41 | 0.23 | 0.083 |
| variable [FCQ_total_post] | 0.75 | 0.17 | **<0.001** |
| condition [stress] | 0.60 | 0.31 | 0.057 |
| drink [sugar] | 0.00 | 0.32 | 0.999 |
| SSES mean | 0.12 | 0.11 | 0.299 |
| variable [FCQ_total_post] × condition [stress] | -0.27 | 0.23 | 0.248 |
| variable [FCQ_total_post] × drink [sugar] | -0.67 | 0.24 | **0.006** |
| condition [stress] × drink [sugar] | -0.19 | 0.44 | 0.677 |
| variable [FCQ_total_post] × SSES mean | 0.16 | 0.08 | 0.066 |
| (variable [FCQ_total_post] × condition [stress]) × drink [sugar] | -0.01 | 0.33 | 0.981 |
| **Random Effects** | | | |
| σ^2^ | 0.08 | | |
| τ_00_ _code_ | 0.62 | | |
| τ_11_ _code.variableFCQ_total_post_ | 0.22 | | |
| ρ_01_ _code_ | -0.01 | | |
| ICC | 0.90 | | |
| N _code_ | 62 | | |
| Observations | 124 | | |
| Marginal R^2^ / Conditional R^2^ | 0.194 / 0.919 | | |

***Table S8.*** Evaluation of the model in which we predicted Food Craving by cortisol reactity (AUCi), glucose reactivity (AUCi) and time (2 levels: pre, post).

|  | **value** | | |
| --- | --- | --- | --- |
| *Predictors* | *Estimates* | *std. Error* | *p* |
| (Intercept) | -0.12 | 0.12 | 0.300 |
| variable [FCQ_total_post] | 0.27 | 0.08 | **0.002** |
| AUCi cort | 0.05 | 0.12 | 0.660 |
| AUCi glucs | -0.03 | 0.12 | 0.771 |
| variable [FCQ_total_post] × AUCi cort | -0.18 | 0.09 | **0.037** |
| variable [FCQ_total_post] × AUCi glucs | -0.29 | 0.09 | **0.001** |
| AUCi cort × AUCi glucs | -0.12 | 0.12 | 0.310 |
| (variable [FCQ_total_post] × AUCi cort) × AUCi glucs | 0.07 | 0.08 | 0.413 |
| **Random Effects** | | | |
| σ^2^ | 0.09 | | |
| τ_00_ _code_ | 0.67 | | |
| τ_11_ _code.variableFCQ_total_post_ | 0.23 | | |
| ρ_01_ _code_ | 0.04 | | |
| ICC | 0.90 | | |
| N _code_ | 62 | | |
| Observations | 124 | | |
| Marginal R^2^ / Conditional R^2^ | 0.101 / 0.914 | | |

***Table S9.*** Evaluation of the model in which we predicted Food Craving by cortisol reactity (AUCi), glucose reactivity (AUCi) and time (2 levels: pre, post) while controlling for stress eating tendency (SSES).

|  | **value** | | |
| --- | --- | --- | --- |
| *Predictors* | *Estimates* | *std. Error* | *p* |
| (Intercept) | -0.12 | 0.12 | 0.299 |
| variable [FCQ_total_post] | 0.27 | 0.08 | **0.002** |
| AUCi cort | 0.06 | 0.12 | 0.634 |
| AUCi glucs | -0.03 | 0.12 | 0.823 |
| SSES mean | 0.09 | 0.12 | 0.445 |
| variable [FCQ_total_post] × AUCi cort | -0.17 | 0.08 | **0.042** |
| variable [FCQ_total_post] × AUCi glucs | -0.28 | 0.08 | **0.001** |
| AUCi cort × AUCi glucs | -0.11 | 0.12 | 0.330 |
| variable [FCQ_total_post] × SSES mean | 0.17 | 0.08 | **0.047** |
| (variable [FCQ_total_post] × AUCi cort) × AUCi glucs | 0.08 | 0.08 | 0.350 |
| **Random Effects** | | | |
| σ^2^ | 0.08 | | |
| τ_00_ _code_ | 0.67 | | |
| τ_11_ _code.variableFCQ_total_post_ | 0.21 | | |
| ρ_01_ _code_ | -0.01 | | |
| ICC | 0.90 | | |
| N _code_ | 62 | | |
| Observations | 124 | | |
| Marginal R^2^ / Conditional R^2^ | 0.138 / 0.917 | | |

***Table S10.*** Evaluation of the model in which we predicted Food Craving by condition (2 levels: stress, control), drink (2 levels: glucose, water) and time (2 levels: pre, post) while controlling for stress eating tendency (SSES) and sex (2 levels: male, female).

|  | **value** | | |
| --- | --- | --- | --- |
| *Predictors* | *Estimates* | *std. Error* | *p* |
| (Intercept) | -0.31 | 0.33 | 0.353 |
| variable [FCQ_total_post] | 1.12 | 0.24 | **<0.001** |
| condition [stress] | 0.26 | 0.42 | 0.541 |
| drink [sugar] | -0.31 | 0.45 | 0.502 |
| sex [male] | -0.16 | 0.47 | 0.732 |
| variable [FCQ_total_post] × condition [stress] | -0.74 | 0.31 | **0.019** |
| variable [FCQ_total_post] × drink [sugar] | -0.74 | 0.33 | **0.028** |
| condition [stress] × drink [sugar] | 0.48 | 0.61 | 0.432 |
| variable [FCQ_total_post] × sex [male] | -0.67 | 0.34 | 0.051 |
| condition [stress] × sex [male] | 0.78 | 0.63 | 0.219 |
| drink [sugar] × sex [male] | 0.58 | 0.64 | 0.370 |
| (variable [FCQ_total_post] × condition [stress]) × drink [sugar] | 0.20 | 0.44 | 0.648 |
| (variable [FCQ_total_post] × condition [stress]) × sex [male] | 0.95 | 0.46 | **0.041** |
| (variable [FCQ_total_post] × drink [sugar]) × sex [male] | 0.09 | 0.46 | 0.853 |
| (condition [stress] × drink [sugar]) × sex [male] | -1.55 | 0.90 | 0.090 |
| (variable [FCQ_total_post] × condition [stress] × drink [sugar]) × sex [male] | -0.54 | 0.65 | 0.407 |
| **Random Effects** | | | |
| σ^2^ | 0.07 | | |
| τ_00_ _code_ | 0.59 | | |
| τ_11_ _code.variableFCQ_total_post_ | 0.20 | | |
| ρ_01_ _code_ | 0.01 | | |
| ICC | 0.90 | | |
| N _code_ | 62 | | |
| Observations | 124 | | |
| Marginal R^2^ / Conditional R^2^ | 0.231 / 0.925 | | |

***Table S11.*** Evaluation of the model in which we predicted Food Craving by cortisol reactity (AUCi), glucose reactivity (AUCi) and time (2 levels: pre, post) while controlling for stress eating tendency (SSES) and sex (2 levels: male, female).

|  | **value** | | |
| --- | --- | --- | --- |
| *Predictors* | *Estimates* | *std. Error* | *p* |
| (Intercept) | -0.09 | 0.17 | 0.621 |
| variable [FCQ_total_post] | 0.37 | 0.12 | **0.004** |
| AUCi cort | 0.25 | 0.22 | 0.253 |
| AUCi glucs | -0.16 | 0.16 | 0.317 |
| sex [male] | 0.05 | 0.26 | 0.832 |
| variable [FCQ_total_post] × AUCi cort | -0.21 | 0.15 | 0.182 |
| variable [FCQ_total_post] × AUCi glucs | -0.19 | 0.11 | 0.093 |
| AUCi cort × AUCi glucs | -0.25 | 0.18 | 0.175 |
| variable [FCQ_total_post] × sex [male] | -0.31 | 0.18 | 0.091 |
| AUCi cort × sex [male] | -0.37 | 0.33 | 0.266 |
| AUCi glucs × sex [male] | 0.27 | 0.25 | 0.279 |
| (variable [FCQ_total_post] × AUCi cort) × AUCi glucs | 0.03 | 0.13 | 0.790 |
| (variable [FCQ_total_post] × AUCi cort) × sex [male] | 0.31 | 0.23 | 0.175 |
| (variable [FCQ_total_post] × AUCi glucs) × sex [male] | -0.32 | 0.17 | 0.076 |
| (AUCi cort × AUCi glucs) × sex [male] | 0.03 | 0.35 | 0.922 |
| (variable [FCQ_total_post] × AUCi cort × AUCi glucs) × sex [male] | 0.41 | 0.25 | 0.104 |
| **Random Effects** | | | |
| σ^2^ | 0.08 | | |
| τ_00_ _code_ | 0.65 | | |
| τ_11_ _code.variableFCQ_total_post_ | 0.20 | | |
| ρ_01_ _code_ | 0.09 | | |
| ICC | 0.91 | | |
| N _code_ | 62 | | |
| Observations | 124 | | |
| Marginal R^2^ / Conditional R^2^ | 0.131 / 0.922 | | |

***Figure S3.*** Correlation matrix depicting Spearman’s correlation coefficients between cortisol and glucose reactivity and changes in facets of food craving. Statistically significant coefficients that survive Bonferroni correction for 11 tests (alpha_adj_ = .005) are shaded with a colored circle (blue = positive association, red = negative association).
